# RPA and XPA interaction with DNA structures mimicking intermediates of the late stages in nucleotide excision repair

**DOI:** 10.1371/journal.pone.0190782

**Published:** 2018-01-10

**Authors:** Yuliya S. Krasikova, Nadejda I. Rechkunova, Ekaterina A. Maltseva, Olga I. Lavrik

**Affiliations:** 1 Institute of Chemical Biology and Fundamental Medicine, Novosibirsk, Russia; 2 Department of Natural Sciences, Novosibirsk State University, Novosibirsk, Russia; University of South Alabama Mitchell Cancer Institute, UNITED STATES

## Abstract

Replication protein A (RPA) and the xeroderma pigmentosum group A (XPA) protein are indispensable for both pathways of nucleotide excision repair (NER). Here we analyze the interaction of RPA and XPA with DNA containing a flap and different size gaps that imitate intermediates of the late NER stages. Using gel mobility shift assays, we found that RPA affinity for DNA decreased when DNA contained both extended gap and similar sized flap in comparison with gapped-DNA structure. Moreover, crosslinking experiments with the flap-gap DNA revealed that RPA interacts mainly with the ssDNA platform within the long gap and contacts flap in DNA with a short gap. XPA exhibits higher affinity for bubble-DNA structures than to flap-gap-containing DNA. Protein titration analysis showed that formation of the RPA-XPA-DNA ternary complex depends on the protein concentration ratio and these proteins can function as independent players or in tandem. Using fluorescently-labelled RPA, direct interaction of this protein with XPA was detected and characterized quantitatively. The data obtained allow us to suggest that XPA can be involved in the post-incision NER stages via its interaction with RPA.

## Introduction

Organism survival depends on efficiently maintaining the integrity of the genome, such as accurate replication of base pairs as well as identifying and repairing a variety of DNA lesions. Nucleotide excision repair (NER) is the only mechanism in humans that can repair double helix-distorting lesions, such as UV-induced pyrimidine dimers or bulky chemical adducts caused by environmental carcinogens or chemotherapeutic agents. NER operates through two distinct pathways: global genome NER (GG-NER) and transcription coupled NER (TC-NER), where the lesion-stalled RNA polymerase II triggers the assembly of the repair machinery at the site of damage [1, 2]. The minimal set of proteins required to perform complete GG-NER (more than 30 polypeptides) was established using an *in vitro* reconstituted system [3].

The currently accepted hypothesis is that the lesions are initially recognized either by the heterotrimeric xeroderma pigmentosum group C (XPC) protein complex (composed of XPC, RAD23B, and Centrin-2) [4, 5] or by the UV-damaged DNA-binding protein (UV-DDB, also known as the DDB1-DDB2 heterodimer or XPE factor), which detects UV-induced DNA photo-lesions and facilitates XPC loading [6, 7]. Then, the XPC complex recruits the transcription factor II (TFIIH). The XPD helicase from TFIIH complex partially opens the DNA helix with its 5' to 3' unwinding activity and provides damage verification by stalling at the site of the bulky lesion [8, 9]. The XPD helicase activity is modulated by XPA [10]. The final assembly of the pre-incision complex includes XPA, replication protein A (RPA), and structure-specific endonucleases XPG and XPF-ERCC1 [11]. The first DNA incision 5' to the site of damage is carried out by XPF-ERCC1 and then 3'-incision is carried out by XPG [12]. Following the removal of the damaged oligonucleotide, the gap is filled by the replication machinery, and DNA ligase I or DNA ligase III-XRCC1 seals the remaining nick [13].

RPA, the major eukaryotic ssDNA-binding protein, is essential for DNA replication, recombination, repair, and DNA damage checkpoint. Each of the RPA subunits (70, 32 and 14 kDa) contains one or more DNA-binding domains (DBD) [14, 15] that cause RPA to possess multiple modes of DNA binding that differ in the amount of nucleotide residues occluded by the protein and in the number of domains interacting with the DNA [16–18]. RPA binds ssDNA with high affinity [19, 20] and it also binds to damaged dsDNA with some degree of specificity [21–23].

RPA plays an integral role in NER damage recognition preceding the incision of the damage, and then again in post-excision repair synthesis [24–26]. *In vitro* RPA greatly enhanced binding to artificial DNA structures and stimulated endonuclease activity of XPF-ERCC1 [27, 28] but in XPA-deficient cells XPF-ERCC1 was not recruited to the sites of damage despite the presence of RPA [27, 29]. RPA also interacts with and stimulates XPG [27]. The absence of RPA leads to a nonfunctional pre-incision complex that lacks incision activity [30]. These data suggest that RPA is sufficient for correct orientation and activation of the XPG and XPF-ERCC1, which are recruited by TFIIH and XPA, respectively [11, 27, 28, 29,31,32].

During the NER process the excised 25–30 mer damaged oligonucleotide is released. The size of the excised fragment coincides with maximal length of ssDNA platform for RPA binding (~30 nt), to which RPA binds tightly with a defined 5′→3′ polarity [17, 18, 33, 34]. First, it was proposed that the polarity of this RPA binding mode should be important to coordinate assembly of the excision nucleases [18], but the most recent data revealed that a short DNA binding mode (less than 20 nt) is more significant for repair than binding to a long DNA fragment [35]. In either case, the undamaged strand is probably protected from nuclease attack by RPA [23, 36]. Recently, it was shown that the excised oligonucleotide is released from the chromatin in complex with TFIIH and then is bound by RPA [37]. RPA helps to promote arrival and positioning of RFC [25, 38], and enhances NER-mediated DNA synthesis [39]. A model, wherein RPA regulates NER by allowing initiation of new repair events only after re-synthesis is completed, was recently proposed [13, 30].

The main RPA partner in the NER process is XPA. XPA exhibits significantly higher affinity for branched DNA structures than for linear DNA [40] and interacts with many NER proteins: RPA, XPC, DDB, TFIIH, XPF-ERCC1 and PCNA [41, 42]. Mutations in the XPA gene have a most dramatic effect on the viability of the cell and organism, underscoring the importance of this protein [43]. Taken together, these observations allow suggesting XPA as a central hinge in the NER complex and its loss leads to collapse of the complex.

It was shown recently that XPA promotes TFIIH translocation along the DNA and modulates its helicase activity: XPA activates unwinding of normal DNA and inhibits helicase activity in the presence of a bulky lesion [11]. Together with the earlier reported specific affinity of XPA for modified nucleotides in an ssDNA context, this study uncovered the role of XPA in NER as a damage verification factor. Moreover, XPA interacts with the XPF-ERCC1 endonuclease and provides its precise positioning in the pre-incision complex [41]. Taking in account a recent report on the direct interaction of XPA with PCNA [44], questions regarding the implication of XPA in the post-incision stages and its function at the late NER stages arise.

We have previously shown that RPA and XPA effectively bind to DNA structures containing an extended bubble that imitates the DNA intermediate of the pre-incision stage in the NER process [23]. RPA can recognize the bulky lesion in the bubble and interacts mainly with the undamaged strand whereas XPA interacts preferably with the ss/ds DNA junction 5′ of a lesion. This positioning of RPA and XPA can promote loading of incision endonucleases to the NER complex in the proper orientation.

To further understand the roles of RPA and XPA in the NER process, we analyzed the interaction of these proteins with DNA structures imitating intermediates of the late stages of the repair process. We have used 60-mer DNA duplexes containing a 31 nt flap with the fluorescein substituted dUMP residue mimicking the bulky lesion and a 26 or 10 nt gap. The structure containing a damaged flap and a 26 nt gap imitates the DNA intermediate arising in the NER process after damaged strand cleavage by XPF-ERCC1; DNA with the same flap and a 10 nt gap can be attributed to the intermediate of the subsequent partial gap filling [12]. The data obtained support the idea that RPA binds preferably to the undamaged strand and XPA is involved in post-incision NER stages in the complex with RPA.

## Materials and methods

### Reagents and oligonucleotides

**Fig 1:**
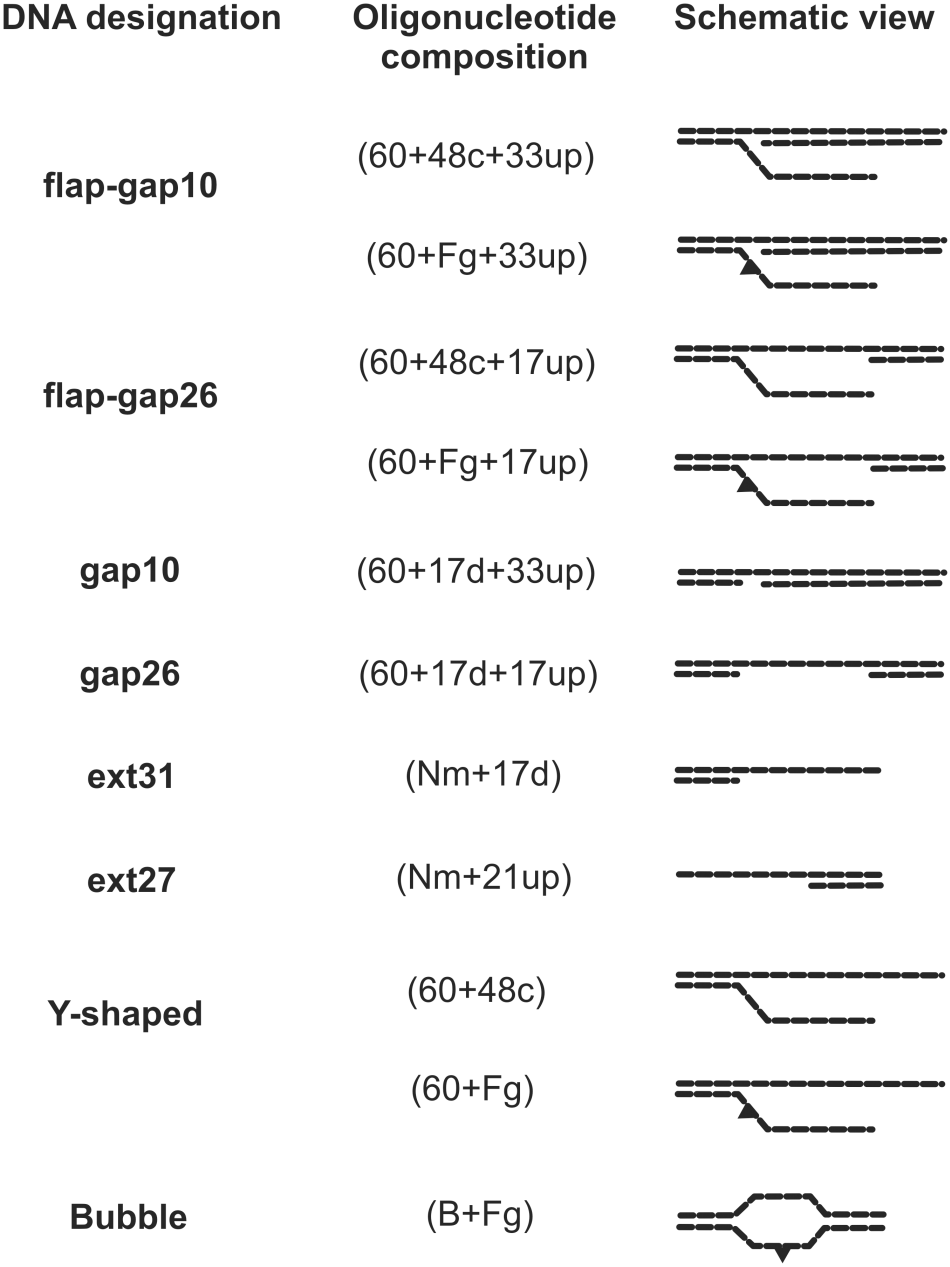
Structures of model DNA used in the study. For oligonucleotide sequences see S1 Table.

[γ-^32^P]ATP (3000 Ci/mmol) was produced in the Laboratory of Biotechnology (Institute of Chemical Biology and Fundamental Medicine, Novosibirsk); phage T4 polynucleotide kinase was purchased from Biosan (Russia); stained molecular mass markers were from BioRad (USA), reagents for electrophoresis and buffer components were from Sigma (USA). The oligonucleotides bearing a fluorescein dUMP derivative (Flu-dUMP, 5-{3-[6-(carboxyamido-fluoresceinyl)amidocapromoyl]allyl}-dUMP) and/or 5-iodo-dUMP (5I-dUMP) were custom-synthesized by Nanotech-C (Russia). A schematic view of the DNA structures is presented in Fig 1; the sequences of the oligonucleotides are presented in S1 Table.

### Protein purification

Recombinant human RPA was purified from *Escherichia coli* as indicated [45]. The plasmid containing the cDNA of hRPA was a kind gift of Dr. M.S. Wold (Department of Biochemistry, Carver College of Medicine, University of Iowa, Iowa City, USA). Recombinant hXPA bearing the N-terminal polyhistidine fragment was expressed in the *E*. *coli* BL21(DE3)LysS strain, using the pETI5b-XPA recombinant plasmid kindly provided by Dr. O. Schärer (SUNY Stony Brook, USA). Protein purification was performed according to [46] with one modification: EDTA was not added during purification. Coomassie-stained SDS-polyacrylamide gels with purified RPA and XPA are presented in S1 Fig.

The RPA concentration in the sample was normalized to its ssDNA binding activity. The XPA concentration in the sample was used as total protein concentration except in experiments with fluorescently labeled RPA where the XPA concentration was normalized to its activity in DNA binding determined as described [47].

### RPA fluorescent labeling

For RPA labeling, the method described previously [48] was used with modifications in protein separation from the reagents. The 5(6)-carboxyfluorescein N-hydroxysuccinimide ester (NHS ester) was dissolved in dimethyl sulfoxide to a final concentration of 10 mM. The reaction mixture (60 μL) contained 12 μL of 10 mM NHS ester, 30 μL of 30 μM RPA in buffer I (100 mM NaCl, 30 mM HEPES (pH 7.8), 0.25 mM EDTA, 10% glycerol, 0.01% NP40). The reaction mixture was incubated overnight at 4°C and then conjugates were separated from the reagents by gel permeation chromatography on Sephadex G-25 Superfine (GE-Healthcare). The chromatography buffer solution contained buffer I with 7 mM 2-mercaptoethanol. All fractions were collected and analyzed by electrophoresis according to Laemmli [49]. The peak fraction containing the protein of interest was used. Fluorescence measurements were performed using POLARstar OPTIMA multifunctional microplate reader (BMG LABTECH GmbH, Germany). The concentration and stoichiometry of the fluorescein (FAM)–RPA conjugates (Flu–RPA) were determined using the extinction coefficients: ε_280_ = 88000 cm^−1^ M^−1^ for RPA, ε_280_ = 23400 cm^−1^ M^−1^ and ε_495_ = 68000 cm^−1^ M^−1^ for FAM (see S2 Fig for Flu-RPA characterization). The Flu-RPA concentration in the sample served as total protein concentration.

### Preparation of 5′-^32^P-labeled DNA

Radioactive label was inserted into the 5′-end of oligonucleotides using phage T4 polynucleotide kinase as described [50]. Labeled oligonucleotides were purified using MicroSpinTM G-25 columns (Amersham Pharmacia Biotech, USA) or by electrophoresis under denaturing conditions followed by passive elution with 3 M LiClO_4_ and acetone precipitation.

The efficiency of oligonucleotide hybridization and the stability of newly synthesized DNA structures were determined by titration of template-primer DNA duplexes by increasing the amounts of oligonucleotide used for the flap strand (S3 Fig). More than 95% DNA were hybridized at a one-to-one ratio of template-primer DNA (60+33up) to flap strand (Fg) (S3 Fig; see S1 Table for oligonucleotide designation and sequences). This ratio was applied for the annealing of all the DNA structure.

DNA structures were prepared by the annealing two 5′-^32^P-labeled oligonucleotides with a complementary oligonucleotide. The oligonucleotides were incubated for 5 min at 95°C, cooled slowly to 75°C, kept for 15 min at this temperature, then cooled slowly to 55°C, kept for 15 min at this temperature, and finally slowly cooled to room temperature. The degree of hybridization was monitored by electrophoresis in a 10% polyacrylamide gel (acrylamide/bis-acrylamide = 40:1) at 4°C. TBE buffer (50 mM Tris, 50 mM H_3_BO_3_, 1 mM EDTA, pH 8.3) was used as the electrode buffer. All DNA structures displayed a high yield and were stable in these conditions (S3 Fig).

### Electrophoretic mobility shift assay

Protein-DNA complexes were analyzed by gel retardation. The reaction mixtures (20 μl) containing buffer A (50 mM Tris-HCl, pH 7.5, 100 mM KCl, 1 mM dithiothreitol, 0.6 mg/ml BSA), 10 nM 5′-^32^P-labeled DNA, or 10 nM fluorescent DNA and proteins at the indicated concentrations were incubated for 20 min at 37°C. Then, 0.2 volume of loading buffer (20% glycerol, 0.015% bromophenol blue in buffer A) at 37°C was added. The samples were loaded onto a 5% native polyacrylamide gel (7% for Flu-RPA-containing samples; acrylamide/bis-acrylamide = 60:1) and separated by electrophoresis in TBE at 4°C and a voltage decrease of 10–12 V/cm. The gels were dried and exposed to storage with a phosphor screen overnight. The positioning of the radioactively labeled oligonucleotide and protein–nucleic acid complexes and quantification of their radioactivity were carried out using a Typhoon FLA 9500 (GE-Healthcare) and Quantity One software. In the case of fluorescein-containing DNA or Flu-RPA, wet gels were scanned by the FAM mode.

### Photoaffinity labeling

Protein modification by photoreactive DNA structures containing a photoreactive 5-iodo-dUMP residue was performed in reaction mixtures (20 μl) containing 50 mM Tris-HCl, pH 7.5, 100 mM KCl, 1 mM dithiothreitol, 0.6 mg/ml BSA, 10 nM 5′-^32^P-labeled photoreactive DNA duplex, and RPA at the specified concentrations. The mixtures were incubated for 30 min at 37°C and then UV-irradiated for 1 h in an ice bath using a Bio-Link BLX-312 cross-linker from Vilber Lourmant (France), wavelength 312 nm, light intensity 5 mJ/cm^2^·sec. The reaction was terminated by 1:5 (v/v) dilution of the sample with stop buffer (5% SDS, 5% 2-mercaptoethanol, 0.3 M Tris-HCl, pH 7.8, 50% glycerol, and 0.005% bromophenol blue). Modified products were separated by electrophoresis according to Laemmli [49] with subsequent autoradiography using the Typhoon FLA 9500. Crosslinking efficiency was estimated as a percentage of radioactivity in the bands corresponding to the modified product related to the total radioactivity in the lane.

### Statistical analysis

All experiments were performed at least in triplicate. The data obtained were quantified using Quantity One software and analyzed with MS Excel. EMSA and crosslinking data are displayed as means+SD.

## Results

### RPA binding to DNA containing a flap and a 26 or 10 nt gap

**Fig 2:**
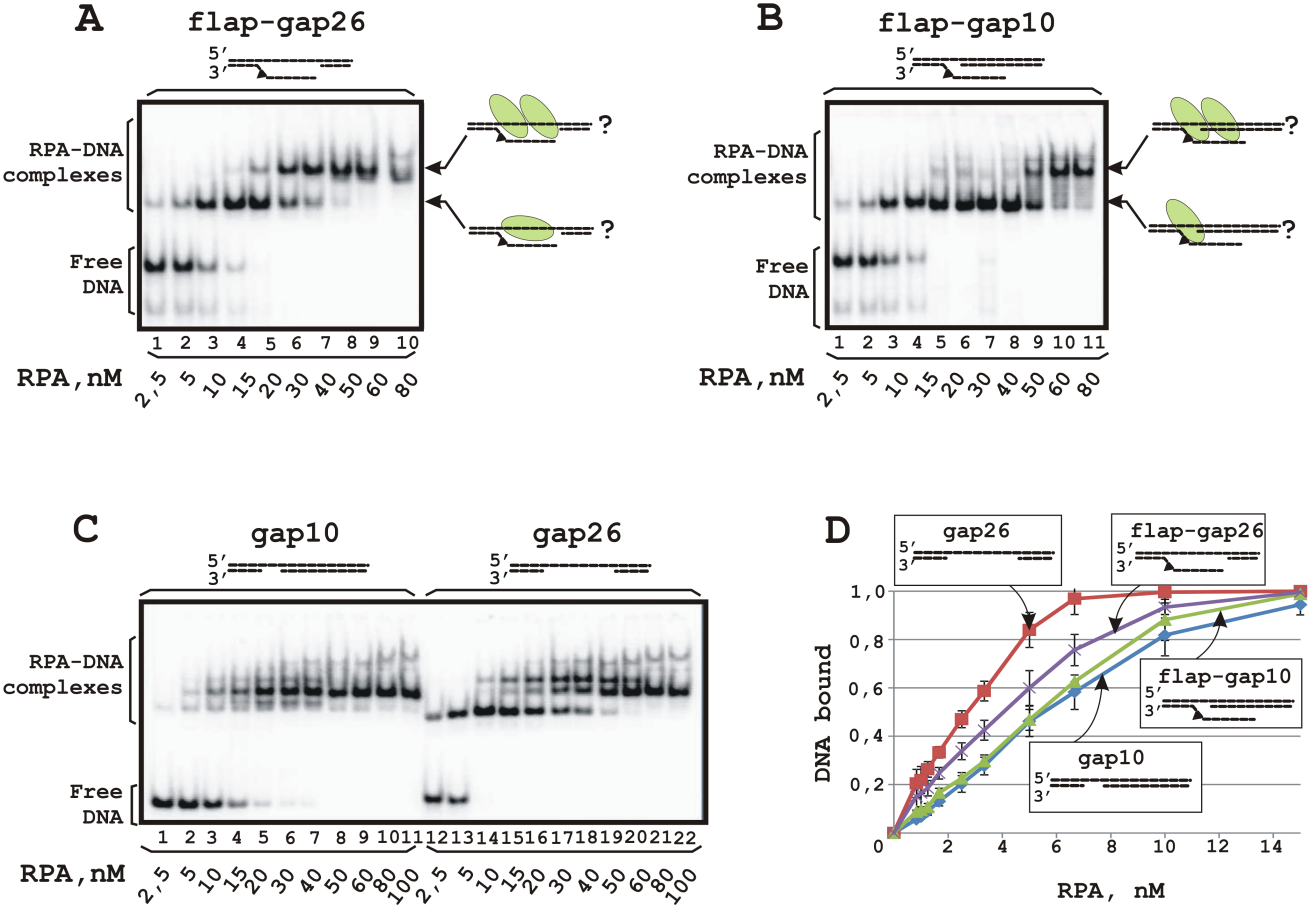
RPA binding to DNA containing a flap with a gap (A, B) or a gap only (C). The reaction mixtures (10 μl) contained buffer A, 10 nM 5′-^32^P-labeled DNA and RPA at the indicated concentrations. A schematic view of the DNA structures is presented at the top: a triangle indicates the position of the bulky lesion. (**A**) Increasing amounts of RPA were added to DNA containing a 26 nt gap and a flap. (**B**) RPA was added to DNA containing a 10 nt gap and a flap. (**C**) RPA was added to DNA containing only gaps: a 10 nt gap (lanes 1–11) or a 26 nt gap (lanes 12–22). (**D**) Summary plot of the data of the binding experiments shown in panels **A**-**C**. Averages and standard deviations were estimated from three independent experiments.

RPA in globular conformation binds a short 8–10 nt ssDNA platform [45, 51] and this binding mode is significant for repair [35]. Potentially, with an excess of protein, at least 4 RPA molecules can bind to flap–gap26 DNA (two with the gap and the other two–with the flap). Therefore, DNA titration by increasing the amount of RPA should result in the sequential appearance of complexes corresponding to different amounts of bound RPA molecules–from 1 to 4 and more. Surprisingly, only two major complexes were observed in the EMSA experiments (Fig 2A). The first corresponds to binding of the single RPA molecule to the DNA (lanes 1–3, Fig 2A). When the RPA to DNA ratio was more than 1:1, a complex with a slower mobility was visible (lanes 4–10, Fig 2A). This complex became major when RPA was present in more than 2 fold excess to DNA and can be attributed to binding of the second RPA molecule to the RPA-DNA complex.

We also examined RPA interaction with DNA containing a flap and a 10 nt gap (Fig 2B). For this DNA structure one major complex with RPA was observed even in the presence of excess protein (lanes 1–9, Fig 2B). The second band that probably corresponds to the complex with 2 RPA molecules became prevalent at high RPA excess (lanes 10, 11). Although RPA binding profiles to DNA with damaged and undamaged flap were very similar, RPA demonstrated some preference for DNA with a damaged flap, that was more pronounced in the case of DNA with a 10 nt gap (S4 Fig).

**Fig 3:**
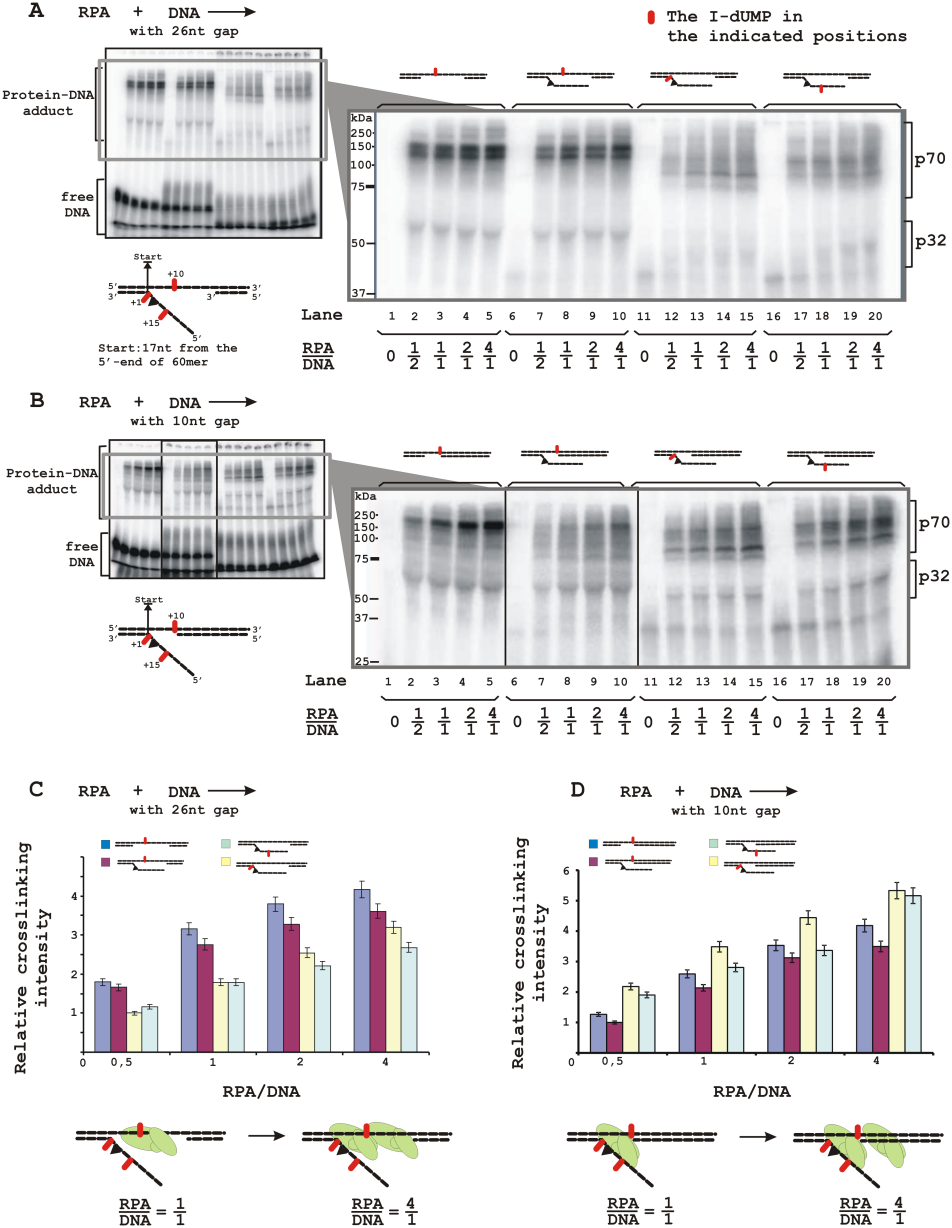
RPA localization on DNA structures containing a damaged flap and a 26 nt gap (A) or a 10 nt gap (B). The reaction mixtures (20 μl) contained buffer A, 10 nM 5′-^32^P-labeled photoreactive DNA containing 5I-dUMP within gap (lanes 1–10) or flap (lanes 11–20) and RPA at the indicated concentrations. A schematic view of the DNA structures is presented: a triangle indicates the position of the bulky lesion, dashes and numbers indicate the position of 5I-dUMP relative to the start (ss/ds DNA junction). The photocrosslinking products were separated by SDS-PAGE and visualized by autoradiography. (**C**) Quantitative analysis of the data from (A) and (B) for the p70 subunit. Averages and standard deviations were estimated from three independent experiments. The bottom panel presents a schematic view of the RPA localization on the DNA based on maximum photocrosslinking intensity.

To reveal the impact of the flap in the affinity of RPA for DNA, we compared protein binding to flap–gap and gap containing DNA structures. As expected, RPA has a higher affinity for longer gaps (Fig 2C). Moreover, RPA binds gap26 DNA with higher affinity than the DNA containing both the 26 nt gap and the flap, but demonstrates a very similar affinity for DNA containing a 10 nt gap with and without flap (Fig 2D and S4 Fig). RPA also demonstrates a difference in the binding mode to various sized gaps–it forms one major complex with the duplex containing a 10 nt gap (lanes 1–11, Fig 2C) and 2–3 complexes with the duplex containing a 26 nt gap (lanes 12–22, Fig 2C). Interestingly, RPA complexes with the gap26 DNA demonstrate aberrant mobility in the gel: the band with lower mobility is observed at lower protein concentrations (lanes 14, 15). Further titration results in the third band with higher mobility that becomes major at RPA to DNA ratios of more than 5 (lanes 20–22) and moves similarly to the RPA complex with gap10 DNA (lanes 1–11). It was shown previously that complexes of protein bound to the central part of DNA move more slowly in gels than when complexed with the DNA ends [52]. In the case of gap26 DNA two RPA molecules can bind to the ssDNA platform in the central part of the DNA and this complex moves more slowly than complexes including more protein molecules also bound to DNA ends. It should be noted that such aberrant binding behavior was also observed for flap-gap26 DNA (Fig 2A, lanes 8–10), whereas to all other DNA structures containing extended single-stranded fragments, e.g. Y-shaped, 5′- or 3′-overhang DNA structures (S5 Fig) RPA binds in the regular manner. Based on these results we assume that RPA interacts with the ssDNA platform in the gap rather than with the flap.

### RPA crosslinking with ssDNA in the gap or with the flap

To discriminate the ssDNA platform preferable for RPA binding in the flap-gap DNA, we performed crosslinking experiments using specific DNA structures containing a photoreactive 5I-dUMP residue in a defined position in the gap or flap. Fig 3 presents data on RPA crosslinks to these DNA structures. One can see that RPA crosslinks to DNA mainly via the p70 subunit with crosslinking efficiency depending on the DNA structure and the 5I-dUMP position. The highest yield of crosslinks was observed for DNA containing 5I-dUMP within the 26 nt gap (Fig 3A, lanes 2–5 and corresponding histogram in C). Crosslinking efficiency decreased slightly in the case of flap-gap-DNA with the same 5I-dUMP position (lanes 7–10) and more significantly when the photoreactive residue was placed in the flap (lanes 12–15 and 17–20; histogram in C). Although efficiencies of RPA crosslinking to 5I-dUMP in the beginning and in the middle part of flap were similar, the patterns of the modified products were different, most likely due to various target amino acids.

In the case of a 10 nt gap containing DNA structures, the efficiency of RPA crosslinking with the 5I-dUMP in the gap decreased significantly in the presence of a flap (Fig 3B, compare lanes 2–5 and 7–10; histograms in D). In contrast to DNA with the long gap, efficiency of RPA crosslinking with the 5I-dUMP in the flap was higher than in the gap (compare lanes 7–10 with lanes 12–15 and 17–20; histograms in D). Despite the fact that RPA demonstrated one major complex with this DNA structure (Fig 2B), several bands with similar intensity that can be attributed to modified p70 were observed, in particular with 5I-dUMP in the middle part of the flap (lanes 17–20) most likely due to flexibility of the flap structure that can contact more than one amino acid residue in the protein.

### XPA binding to the model DNA and formation of the ternary RPA-XPA-DNA complex

**Fig 4:**
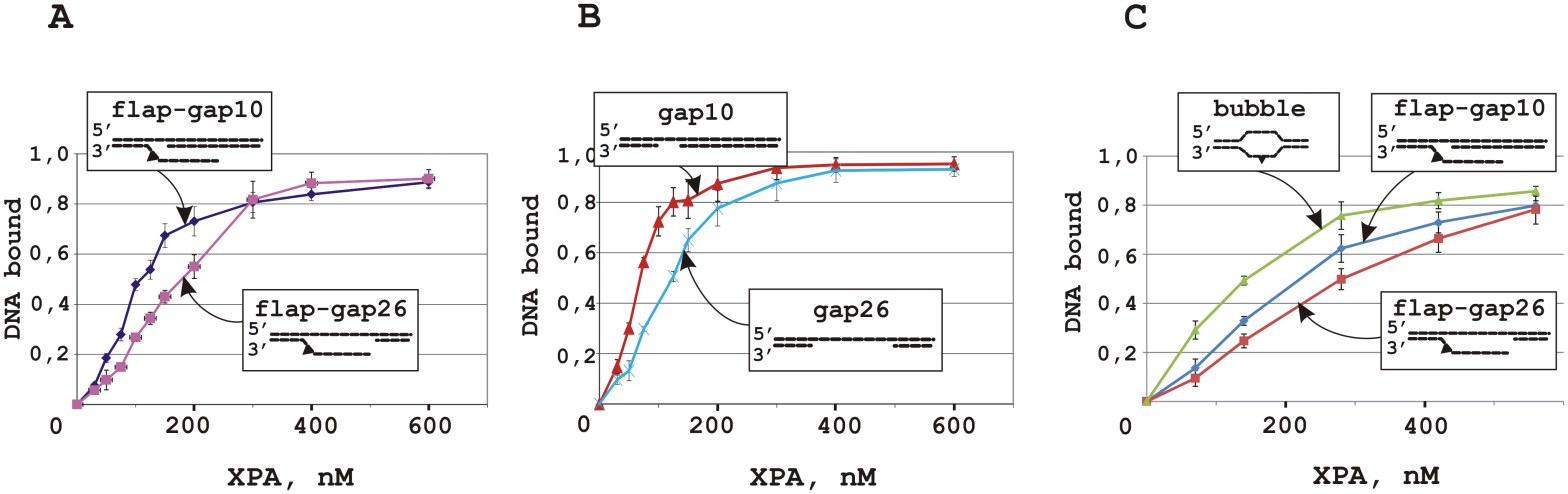
XPA binding to DNA containing a 10 or 26 nt gap (A) or both a gap and a flap (B). (C) XPA binds bubble-DNA more effectively than flap-gap containing DNA. Averages and standard deviations were estimated from three independent experiments.

In contrast to RPA, XPA showed about two times higher affinity for DNA containing a short 10 nt gap than for DNA with a 26 nt gap (Fig 4A and S6 Fig). This prevalent binding for the 10 nt gap was the same in the case of flap-gap DNA (Fig 4B and S6 Fig) although XPA affinity to such DNA was lower than to DNA without flap (compare plots in Fig 4A and 4B). Therefore, both proteins demonstrate a preference for a gap-containing DNA in comparison to flap-gap DNA. We have also compared the affinity of XPA for the studied DNA structures and for the bubble-DNA structure, imitating a pre-incision stage intermediate. Interestingly, XPA binds bubble-DNA more effectively than flap-gap containing DNA, in particular flap-gap26 DNA (Fig 4C and S6 Fig). These observations suggest that incision of the damaged DNA strand by XPF-ERCC1 leads to loss of the preferable binding site for XPA resulting in decreased affinity.

**Fig 5:**
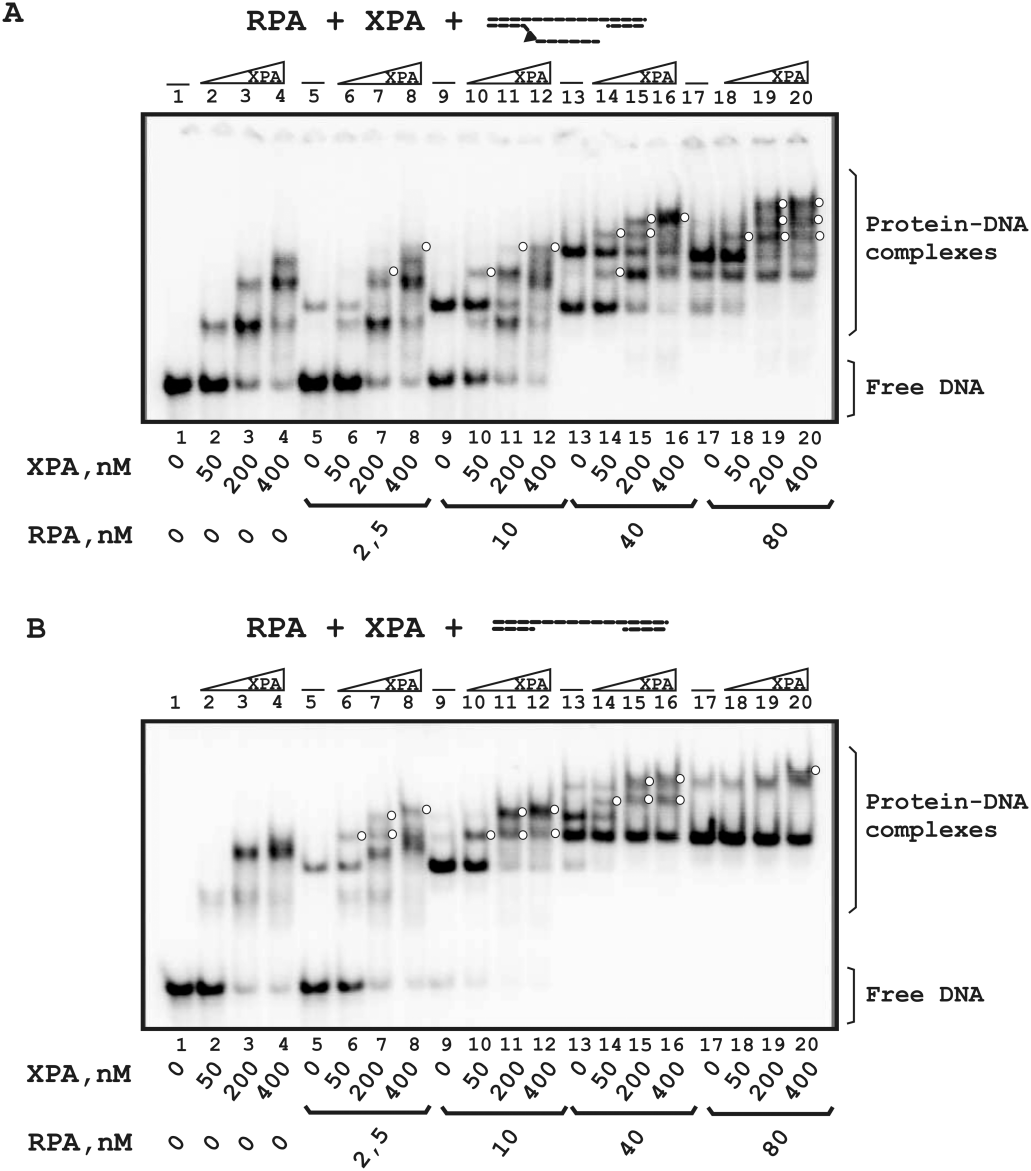
Comparative analysis of RPA and XPA binding to DNA containing the 26 nt gap with the flap (A) or the 26 nt gap only (B). The reaction mixtures (10 μl) contained buffer A, 10 nM 5′-^32^P-labeled DNA and the indicated concentrations of proteins. A schematic view of the DNA structures is presented at the top: a triangle indicates the position of the bulky lesion. White circles indicate putative RPA-XPA-DNA complexes with unknown stoichiometry.

Using various protein:DNA and protein:protein ratios, we analyzed XPA and RPA binding to flap-gap26 (Fig 5A) and gap26 (Fig 5B) DNA. In conditions of RPA deficiency (RPA:DNA = 1:4), two proteins bound separately with flap-gap26 DNA and ternary complexes were not registered (Fig 5A, lanes 2–8). When RPA and DNA concentrations were equal, the intensity of the bands corresponding to XPA and RPA complexes decreased, and a band with lower mobility appeared simultaneously that can be assumed to be the RPA-XPA-DNA complex (compare lane 2 with lanes 9 and 10). Although unbound DNA present in the reaction mixture (lane 9, about a half of the DNA is unbound), free DNA was not used by RPA and XPA for independent binding. In conditions of RPA excess, when DNA was totally complexed with RPA (lanes 13–20), several additional bands were detected that can be assumed to be RPA-XPA-DNA complexes with unknown stoichiometry (for example, two additional bands in lane 14 that correspond neither to RPA-DNA nor to XPA-DNA complexes). It should also be noted that when RPA forms two or more complexes with DNA (lane 13), the RPA-DNA complex with the highest mobility (containing 1 RPA molecule) decreases primarily in the presence of XPA.

As Fig 5B shows, one or two RPA-XPA-DNA complexes are detected with gap26 DNA at each RPA concentration (for example, the bands with the lowest mobility in lanes 6 and 10–12 or additional bands in lanes 14–16). When the RPA and DNA concentrations were equal (lanes 9–12), RPA-DNA complexes were displaced by the ternary complex in the course of titration with XPA. In contrast to flap-gap26 DNA, the intensity of the RPA-DNA complex with the highest mobility does not decrease in the presence of XPA (compare lanes 13–20 in Fig 5A and 5B).

**Fig 6:**
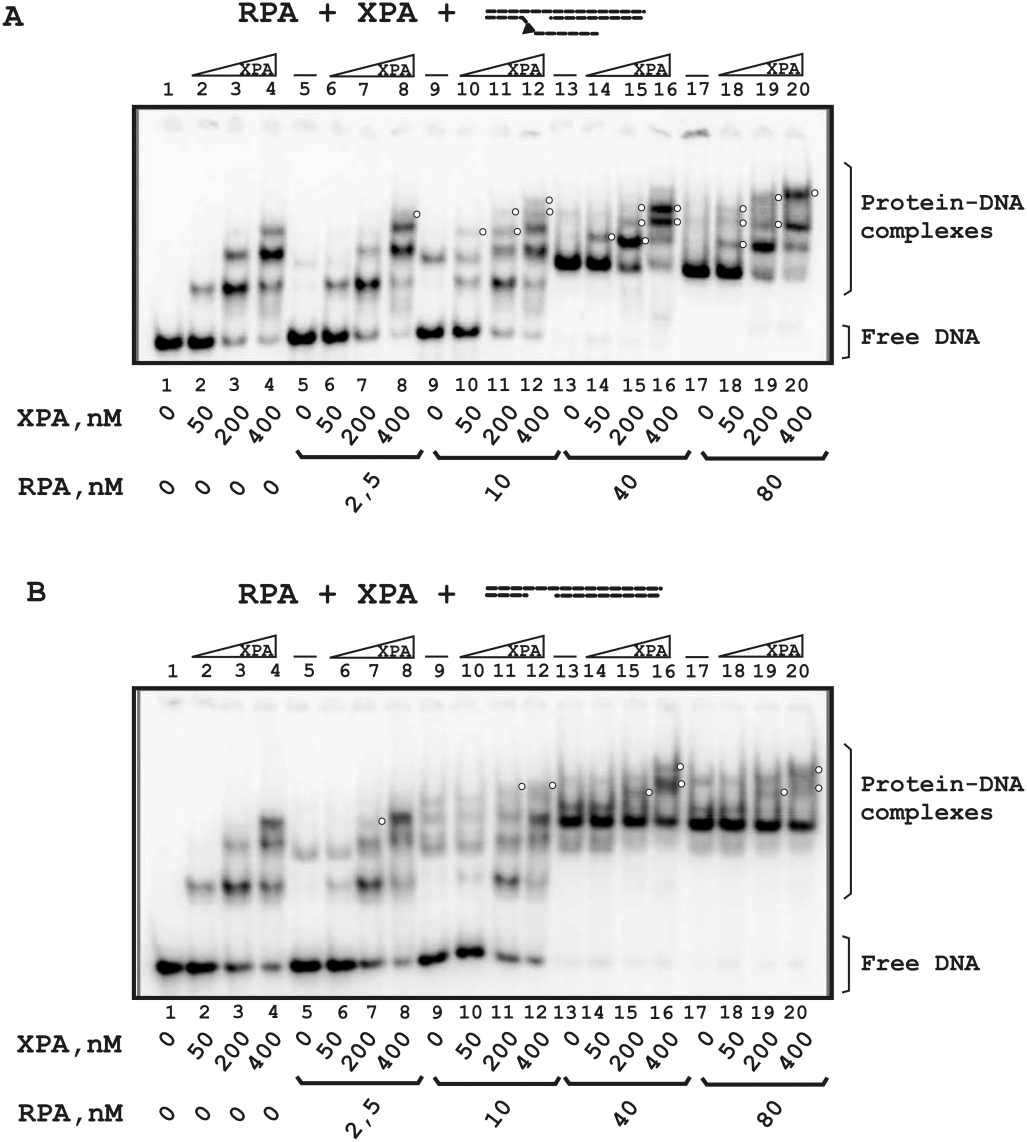
Comparative analysis of RPA and XPA binding to DNA containing a 10 nt gap with a flap (A) or a 10 nt gap only (B). The reaction mixtures (10 μl) contained buffer A, 10 nM 5′-^32^P-labeled DNA and the indicated concentrations of proteins. A schematic view of the DNA structures is presented at the top: a triangle indicates the position of the bulky lesion. White circles indicate putative RPA-XPA-DNA complexes with unknown stoichiometry.

For DNA containing a flap and a 10 nt gap (Fig 6A), only XPA complexes were detected in conditions of RPA deficiency (lanes 2–8). Under equimolar RPA to DNA ratios (lanes 9–12) and in conditions of RPA excess (lanes 13–20), one RPA-XPA-DNA complex was detected (for example, the band with the lowest mobility in lane 16). The ternary complex was more visible for flap-gap10 DNA than for DNA with the 10 nt gap only (compare lanes 17–20 in Fig 6A and 6B). In conditions of RPA deficiency, proteins bound DNA as separate players (lanes 2–12 in Fig 6B). The ternary complex was detected only at high protein concentrations (lanes 16 and 20).

**Fig 7:**
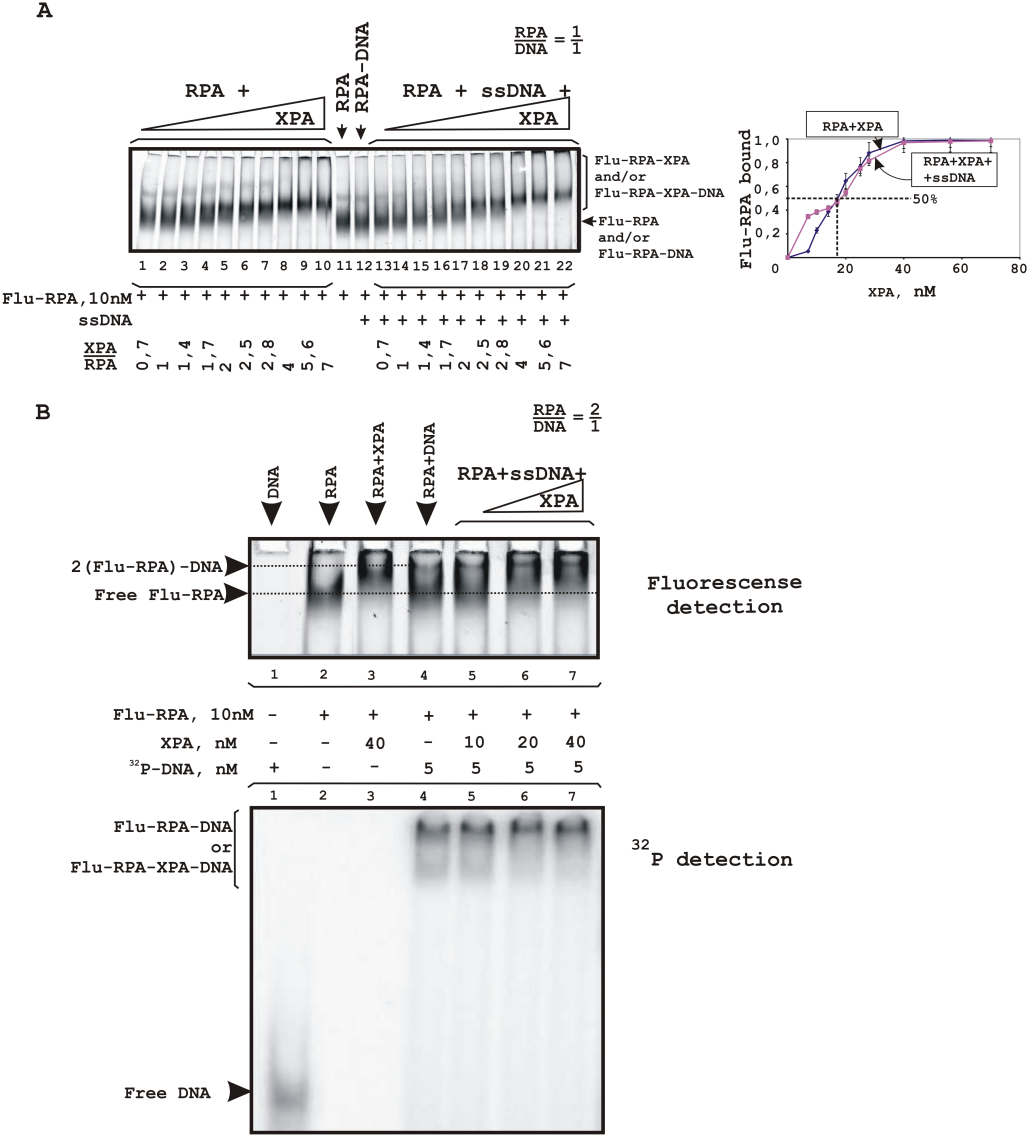
RPA-XPA complex formation. Fluorescently labeled RPA was titrated by XPA in the absence of DNA or in the presence of unlabeled (A) or ^32^P-labeled (B) ssDNA. The reaction mixtures (20 μl) contained buffer A, 10 nM Flu-RPA and indicated concentrations of XPA and/or DNA. A schematic view of the DNA structures is presented at the top: the triangle indicates the position of the bulky lesion. (**A**) The right panel shows the quantitative analysis of the data from the EMSA experiments. Percentage of the XPA protein active in DNA binding was 20%. Averages and standard deviations were estimated from three independent experiments.

To further analyze XPA-RPA interaction, fluorescently labelled proteins were used. Despite the fact that XPA seems to be a simple target for labeling, we were unsuccessful with labeling of this protein and used fluorescent RPA (Flu-RPA). One can see that Flu-RPA molecules enter a native gel in the absence of DNA and their mobility decreases in the presence of XPA (Fig 7A, lanes 1–11). In the presence of ssDNA, differences in mobility between RPA alone and complexed with XPA were more distinct: in the course of titration with XPA, the intensity of the lower band decreased and that of the upper band increased (lanes 12–22). It should be noted that in these experiments we were unable to discriminate free Flu-RPA and its complex with DNA due to the small difference in their molecular masses and to detect a XPA-DNA complex. Using 5′-^32^P-labeled DNA at a concentration that provides a 2 fold excess of Flu-RPA, DNA-protein complexes corresponding most likely to binding of 2 RPA molecules with DNA were visualized (Fig 7B, lane 4) by detection of both fluorescence (upper panel) and radioactivity (lower panel). When increasing amounts of XPA were added, the intensity of the band corresponding to unbound Flu-RPA and/or to the RPA-DNA complex in a 1:1 ratio decreased, whereas the intensity of bands corresponding to the RPA-XPA complex and to the complex with the lowest mobility increased (Fig 7B, upper panel, compare lane 4 with lanes 5–7). According to the ^32^P detection, upper band accumulates almost total DNA in conditions of XPA excess (lanes 6 and 7). These observations allow us to sugges stimulation of Flu-RPA binding to DNA by XPA or binding to DNA of the Flu-RPA-XPA complex. The affinity of XPA for Flu-RPA was determined as the XPA concentration in 50% binding; the estimated EC50 was 18±2 nM (Fig 7A, plot).

**Fig 8:**
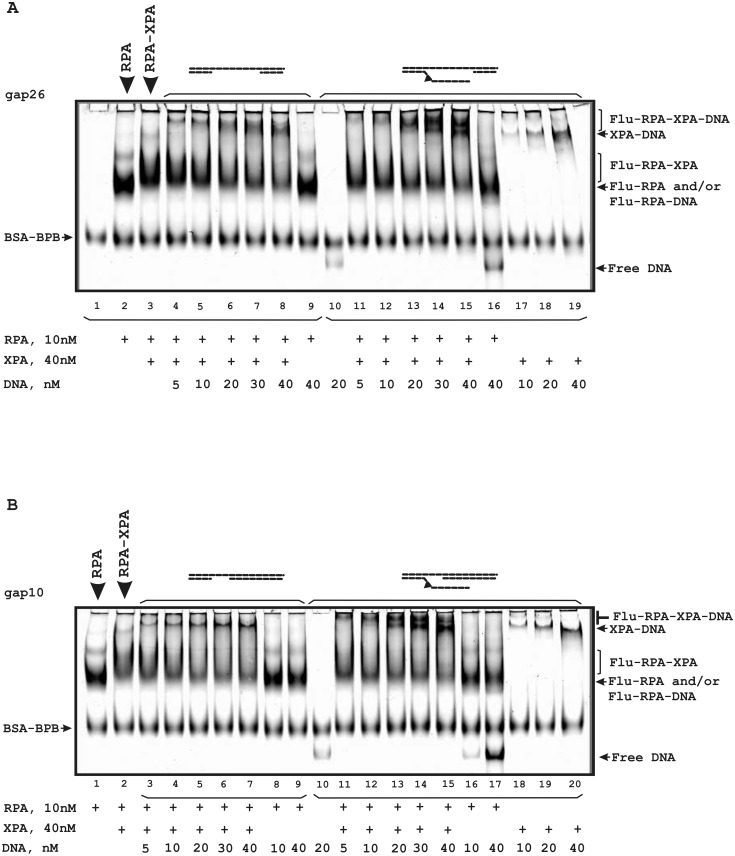
Stability of the RPA-XPA complex in the presence of DNA containing gap/flap-gap26 (A) or gap/flap-gap10 (B). The reaction mixtures (20 μl) contained buffer A, 10 nM Flu-RPA, 40 nM XPA and the indicated concentrations of DNA. A schematic view of the DNA structures is presented at the top: the triangle indicates the position of the bulky lesion. The bands indicated as BSA-BPB correspond to a complex of BSA with bromophenol blue present in the loading buffer.

We also analyzed the influence of gap- and flap-gap-containing DNA on the Flu-RPA interaction with XPA (Fig 8). In the presence of such DNA structures the low mobility bands which correspond to ternary complexes were observed. Moreover, in the case of flap-gap DNA containing the Flu-dUMP residue, additional bands corresponding to XPA-DNA complexes were observed (lanes 11–15 in Fig 8A and 8B). Therefore, XPA and RPA form stable complexes that are not disrupted in the presence of DNA.

## Discussion

Both RPA and XPA are indispensable NER factors required for repair process *in vivo* and *in vitro* [15, 50]. In the present study, we found that XPA binds to DNA mimicking post-incision intermediates with less affinity than to bubble-DNA intermediate (Fig 4C) whereas RPA has some preference for DNA mimicking NER intermediates formed after the action of XPF-ERCC1. These observations provide further evidence for XPA positioning near ss/dsDNA junction in the bubble and allow us to suggest that XPA can be involved in the post-incision NER stages in the complex with RPA.

RPA, an evolutionarily conserved heterotrimeric protein, binds and stabilizes ssDNA regions appearing in various cellular events [14, 15]. In NER, RPA plays an integral role in damage recognition preceding the incision of the damage, and then again in post-excision DNA repair synthesis. RPA polarity appears crucial for the positioning of the excision repair nucleases XPG and XPF–ERCC1 on the DNA [23, 33]. In a previous study we found that RPA interacts with the damaged bubble-DNA structure mainly via binding to the undamaged strand [23]. This position corresponds to the gap region within the flap-gap structure that appears as a result of the action of XPF–ERCC1. Interestingly, in flap-gap26 DNA RPA interacts with the ssDNA track in the gap and does not utilize the flap. Moreover, a long flap impedes RPA binding to DNA, probably by steric hindrance for the protein access to the gap area. It was shown that lesion-containing excised oligonucleotides are released from duplex DNA complexed with TFIIH [37]. Thus, we assume that in NER TFIIH binds to the damaged flap (through its XPD subunit that is stalled on a bulky lesion) and RPA binds to undamaged ssDNA in the gap and protects it from nuclease attack. This mechanism is important for the correct loading of XPG.

**Fig 9:**
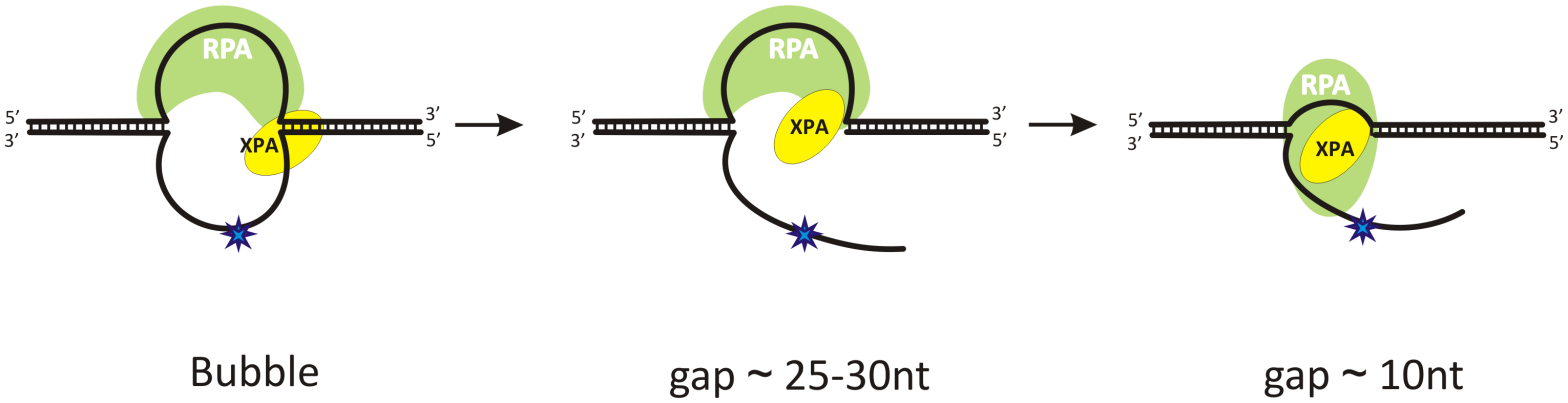
Proposed location of the RPA and XPA proteins in the pre- and post-excision complexes. After XPF-ERCC1 incision, RPA remains bound with the undamaged strand in the gap created. RPA can be translocated on the flap when gap is shortened during the re-synthesis step.

Recently the crystal structure of RPA from *Ustilago maydis* stably bound to ssDNA was resolved [16]. These data revealed that ssDNA complexed with RPA is U-shaped when RPA binds in maximum occluded binding site conformation. The previously proposed “cut-patch-cut-patch” mechanism means that the re-synthesis step starts immediately after the XPF-ERCC1 catalyzed incision [12]. Hence, this raises the question about the possible interaction between factors of pre-incision and re-synthesis steps, especially between participants located at different sides of the pre-incision bubble (for example XPG and PCNA). To combine our data with the results in the literature, we propose that RPA binds to undamaged ssDNA and bends it to the U-shape; this results in pulling together ssDNA ends and makes XPG-PCNA interaction possible [53]. During the re-synthesis step, gap is shortened and this can cause RPA translocation on the flap (Fig 9). We assume that RPA can compete with TFIIH for this binding platform and leave repair assembly in the complex with excised oligonucleotide as was shown previously [37].

The ability of XPA and RPA to form a complex in the absence of DNA as well as a ternary complex with DNA was shown earlier [54, 55] and XPA interaction with RPA is indispensable for NER reaction [56]. Here we visualize such a complex and quantitatively characterize its stability using fluorescently labelled RPA. The estimated affinity of proteins to each other is very similar to that obtained earlier with the surface plasmon resonance biosensor [57] and comparable with the strongest interaction between proteins involved in base excision repair [48]. Moreover, dependence of the ternary RPA-XPA-DNA complex formation on the RPA and XPA concentration indicates that these proteins could function as independent players or as tandem according to different concentration ratios (Fig 5A).

In summary, our results allow us to suggest that within the post-incision NER complex, RPA binds the undamaged DNA strand whereas XPA remains in this complex via interaction with RPA and is placed in the central position that provides an opportunity to contact all other proteins involved in the incision and following re-synthesis stages of NER. Therefore, XPA can be regarded as a key hinge of the protein complex formed on damaged DNA not only in the pre-incision step but also in further NER stages.

## Supporting information

S1 Table Oligonucleotide sequences. The modification is indicated as follows: **F**–Flu-dUMP (fluorescein dUMP derivative (5-{3-[6-(carboxyamido-fluoresceinyl)amidocapromoyl]allyl}-dUMP)); **I–** 5I-dUMP (5-iodo-dUMP).
(DOCX)

S1 Fig Analysis the purified RPA (left panel) and XPA (right panel) proteins in 12.5% SDS-polyacrylamide gels. (TIF)

S2 Fig RPA fluorescent labeling, purification and characterization. (**A**) Analysis of the Flu-RPA fractions by SDS-PAGE followed by fluorescence detection (*left panel*) and coomassie staining (*right panel*). (**B) A**bsorbance spectrum of the central fraction. Number of fluorescein (FAM) molecules conjugated with one RPA molecule was determined from the Flu-RPA absorbance at 280 nm (absorbance of RPA, ε280 = 88000 cm^−1^ M^−1^ and FAM, ε280 = 23400 cm^−1^ M^−1^) and at 495 nm (FAM absorbance, ε495 = 68000 cm^−1^ M^−1^). RPA and FAM concentrations calculated from the presented absorbance spectrum were 5.2*10^−6^ and 1.5*10^−5^ M and number of fluorescein molecules per RPA molecule was 3. (**C**) Analysis of the DNA binding activity of Flu-RPA. The *left panel* presents fluorescence detection. The Flu-RPA-DNA complex has a somewhat higher mobility in the native PAGE than a free Flu-RPA; the 2(Flu-RPA)-DNA complex has lower mobility compared to free Flu-RPA. The *right panel* presents radioactivity detection of DNA in Flu-RPA-DNA complexes. All Flu-RPA molecules were in the complex with DNA and no free DNA was detected at 30 nM Flu-RPA and 6 nM DNA concentrations; therefore 20% of total Flu-RPA was active in DNA binding. (**D**) Analysis of protein binding activity of Flu-RPA. The Flu-RPA-XPA complex has a lower mobility than free Flu-RPA. All Flu-RPA protein turned to the Flu-RPA-XPA complex, so 100% of total Flu-RPA was active in XPA binding. The Flu-RPA concentration in the sample is indicated as a total protein concentration.
(TIF)

S3 Fig Efficiency of the oligonucleotide hybridization (A) and stability of newly synthesized DNA structures in EMSA conditions (B). Template-primer DNA duplexes were titrated by increasing amounts of oligonucleotide used for the flap strand (A). DNA structures formed at a one-to-one ratio of the annealed oligonucleotides were analyzed by electrophoresis in a 10% polyacrylamide gel (B).
(TIF)

S4 Fig Comparative RPA binding to DNA structures containing a 26 nt gap (A) and a 10 nt gap (B). The reaction mixtures (10 μl) contained buffer A, 10 nM 5′-^32^P-labeled DNA and RPA at the indicated concentrations. A schematic view of the DNA structures is presented at the top: the triangle indicates the position of the bulky lesion.
(TIF)

S5 Fig RPA binding to various DNA structures: 5′- or 3′-overhang DNA structures (A) and Y-shaped DNA structures (B). The reaction mixtures (10 μl) contained buffer A, 10 nM 5′-^32^P-labeled DNA and RPA at the indicated concentrations. A schematic view of the DNA structures is presented at the top: the triangle indicates the position of the bulky lesion.
(TIF)

S6 Fig XPA binding to various DNA structures. **(A) DNA containing a flap and a gap, (B) DNA containing a gap only**. The reaction mixtures (10 μl) contained buffer A, 10 nM 5′-^32^P-labeled DNA and XPA at the indicated concentrations. A schematic view of the DNA structures is presented at the top: the triangle indicates the position of the bulky lesion. (**C**) **XPA binds bubble-DNA more effectively than flap-gap containing DNA**. The reaction mixtures (10 μl) contained buffer A, 10 nM Flu-DNA containing flap and 10 nt (lanes 1–6), 26 nt (lanes 7–12) gap or bubble (lanes 13–18) and XPA at the indicated concentrations. In lane 1, the band indicated as BSA-BPB corresponds to a complex of BSA with bromophenol blue that was added to this sample before loading.
(TIF)
