# Supplementary material for: RPA and XPA interaction with DNA structures mimicking intermediates of the late stages in nucleotide excision repair
Source: PLoS One. 2018 Jan 10;13(1):e0190782. doi: 10.1371/journal.pone.0190782 (PMC5761895; doi:10.1371/journal.pone.0190782)
Supplement: S1 Table — The modification is indicated as follows: F–Flu-dUMP (fluorescein dUMP derivative (5-{3-[6-(carboxyamido-fluoresceinyl)amidocapromoyl]allyl}-dUMP)); I– 5I-dUMP (5-iodo-dUMP). (DOCX) [file pone.0190782.s001.docx]

| **Designation** | **Sequences (5'-3')** |
| --- | --- |
| **60** | ctatggcgaggcgattatcaacccatttagtcgtaatagtgaagagtcacgacaacatcg |
| **Photo**  **60** | ctatggcgaggcgattatcaacccat**I**tagtcgtaatagtgaagagtcacgacaacatcg  (+10 position from start in DNA for modification experiments) |
| **Nm** | ctatggcgaggcgattaagttgggсaacgtcagggtcttccgaacgac |
| **48c** | gtcgttcggaagaccctgacgttgcccaacttaatcgcctcgccatag |
| **B** | ctatggcgaggcgattatcaacccattgcagtgggtcttccgaacgac |
| **33up** | cgatgttgtcgtgactcttcactattacgacta |
| **21up** | gtcgttcggaagaccctgacg |
| **17up** | cgatgttgtcgtgactc |
| **17d** | taatcgcctcgccatag |
| **Fg** | gtcgttcggaagaccctgacgt**F**gcccaacttaatcgcctcgccatag |
| **Photo**  **Fg** | gtcgttcggaagaccctgacgt**F**gcccaac**I**taatcgcctcgccatag  (+1 position from start in DNA for modification experiments) |
| **Photo**  **Fg** | gtcgttcggaagaccc**I**gacgt**F**gcccaacttaatcgcctcgccatag  (+15 position from start in DNA for modification experiments) |
