# Supplementary figures and images for: RPA and XPA interaction with DNA structures mimicking intermediates of the late stages in nucleotide excision repair
Source: PLoS One. 2018 Jan 10;13(1):e0190782. doi: 10.1371/journal.pone.0190782 (PMC5761895; doi:10.1371/journal.pone.0190782)

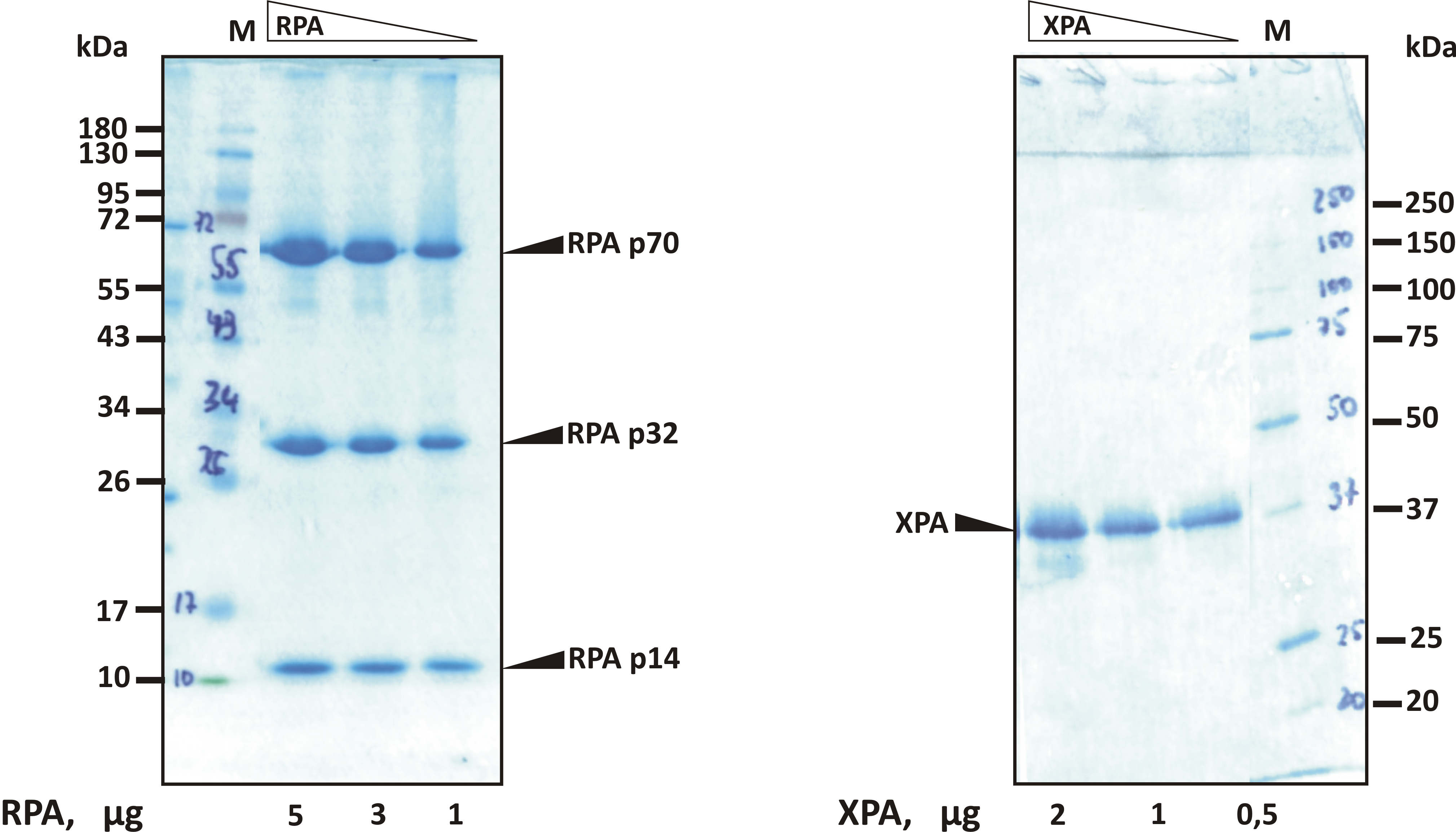

Supplement: S1 Fig — (TIF) [file pone.0190782.s002.TIF]

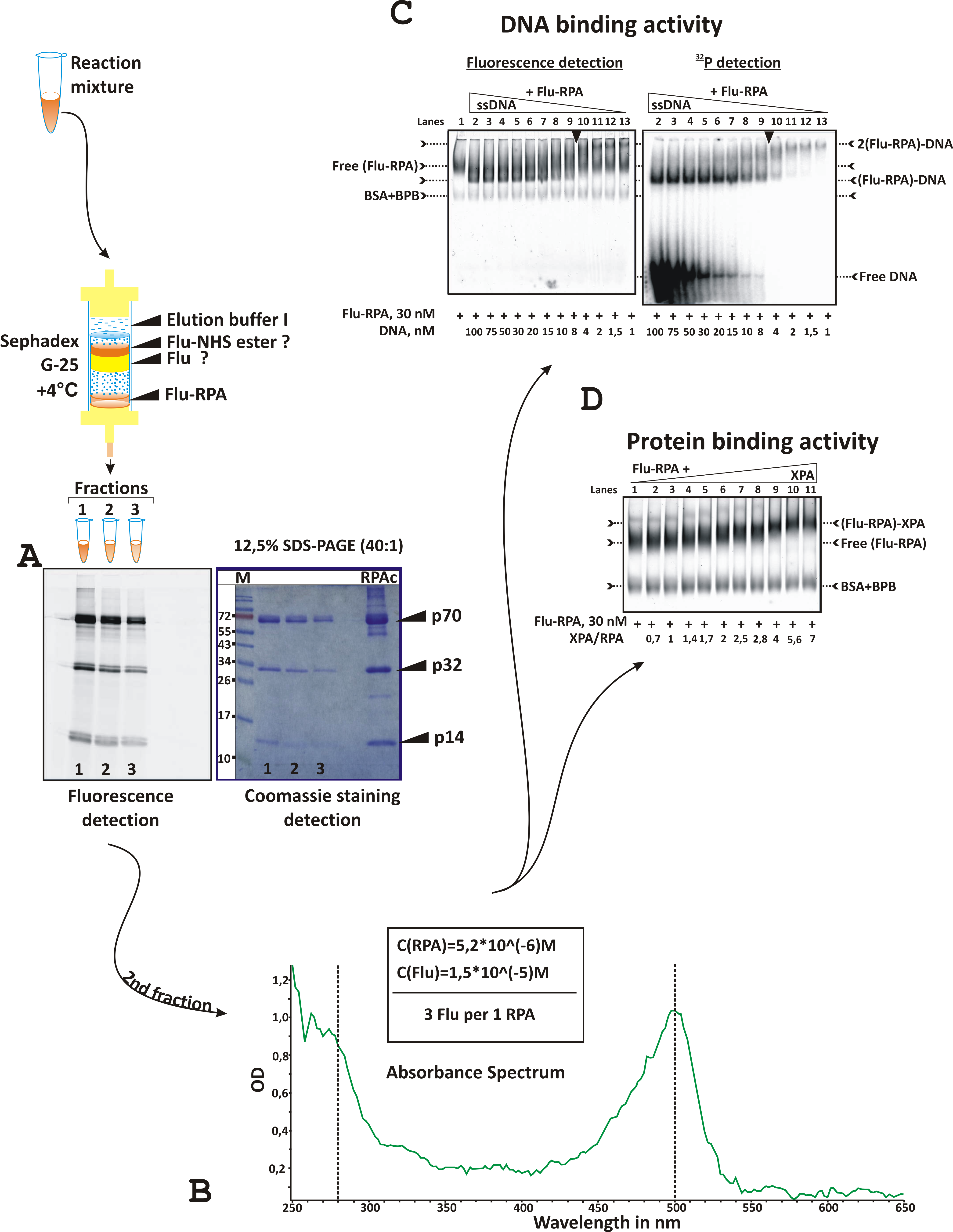

Supplement: S2 Fig — (A) Analysis of the Flu-RPA fractions by SDS-PAGE followed by fluorescence detection (left panel) and coomassie staining (right panel). (B) Absorbance spectrum of the central fraction. Number of fluorescein (FAM) molecules conjugated with one RPA molecule was determined from the Flu-RPA absorbance at 280 nm (absorbance of RPA, ε280 = 88000 cm−1 M−1 and FAM, ε280 = 23400 cm−1 M−1) and at 495 nm (FAM absorbance, ε495 = 68000 cm−1 M−1). RPA and FAM concentrations calculated from the presented absorbance spectrum were 5.2*10−6 and 1.5*10−5 M and number of fluorescein molecules per RPA molecule was 3. (C) Analysis of the DNA binding activity of Flu-RPA. The left panel presents fluorescence detection. The Flu-RPA-DNA complex has a somewhat higher mobility in the native PAGE than a free Flu-RPA; the 2(Flu-RPA)-DNA complex has lower mobility compared to free Flu-RPA. The right panel presents radioactivity detection of DNA in Flu-RPA-DNA complexes. All Flu-RPA molecules were in the complex with DNA and no free DNA was detected at 30 nM Flu-RPA and 6 nM DNA concentrations; therefore 20% of total Flu-RPA was active in DNA binding. (D) Analysis of protein binding activity of Flu-RPA. The Flu-RPA-XPA complex has a lower mobility than free Flu-RPA. All Flu-RPA protein turned to the Flu-RPA-XPA complex, so 100% of total Flu-RPA was active in XPA binding. The Flu-RPA concentration in the sample is indicated as a total protein concentration. (TIF) [file pone.0190782.s003.TIF]

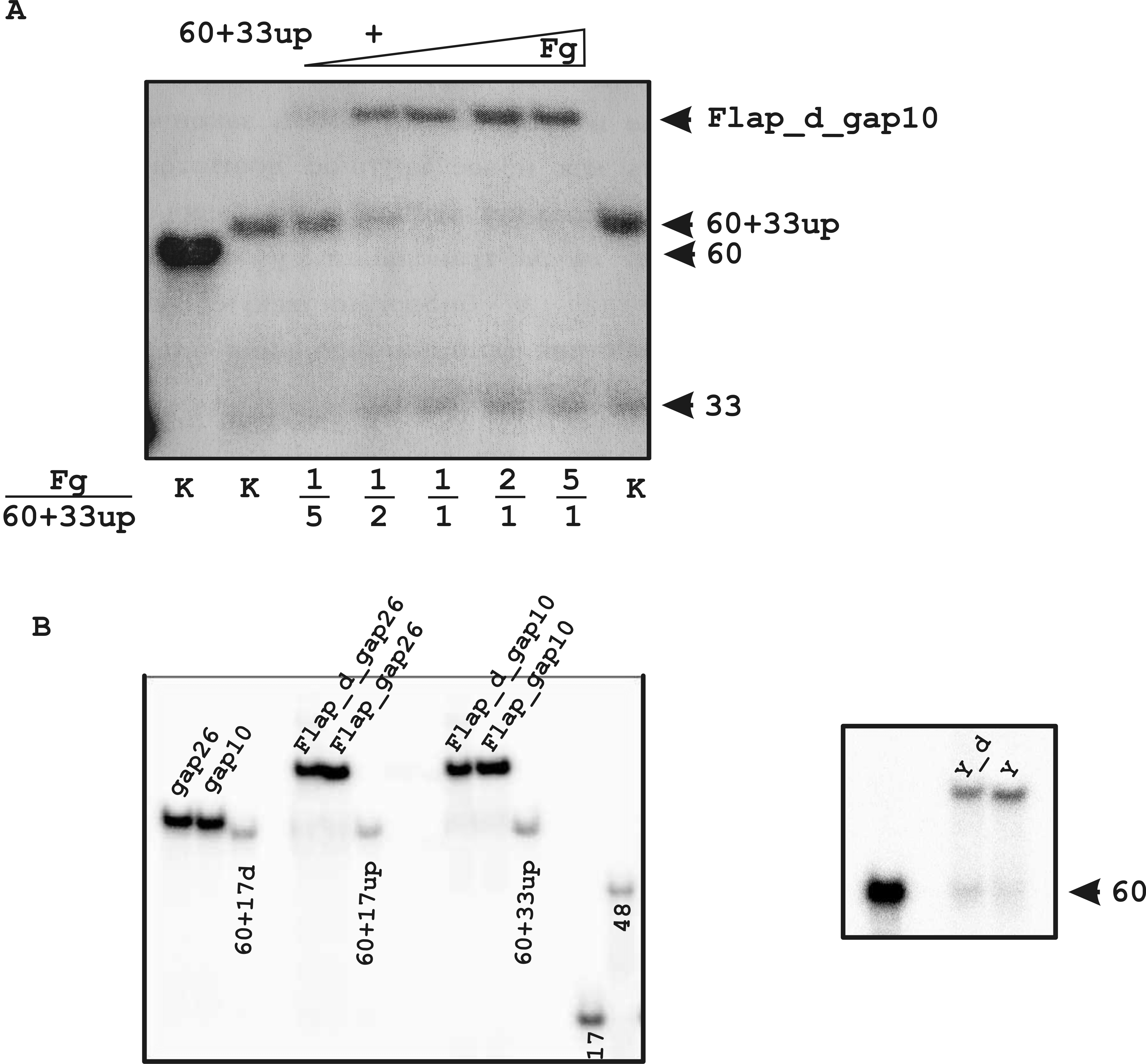

Supplement: S3 Fig — Template-primer DNA duplexes were titrated by increasing amounts of oligonucleotide used for the flap strand (A). DNA structures formed at a one-to-one ratio of the annealed oligonucleotides were analyzed by electrophoresis in a 10% polyacrylamide gel (B). (TIF) [file pone.0190782.s004.TIF]

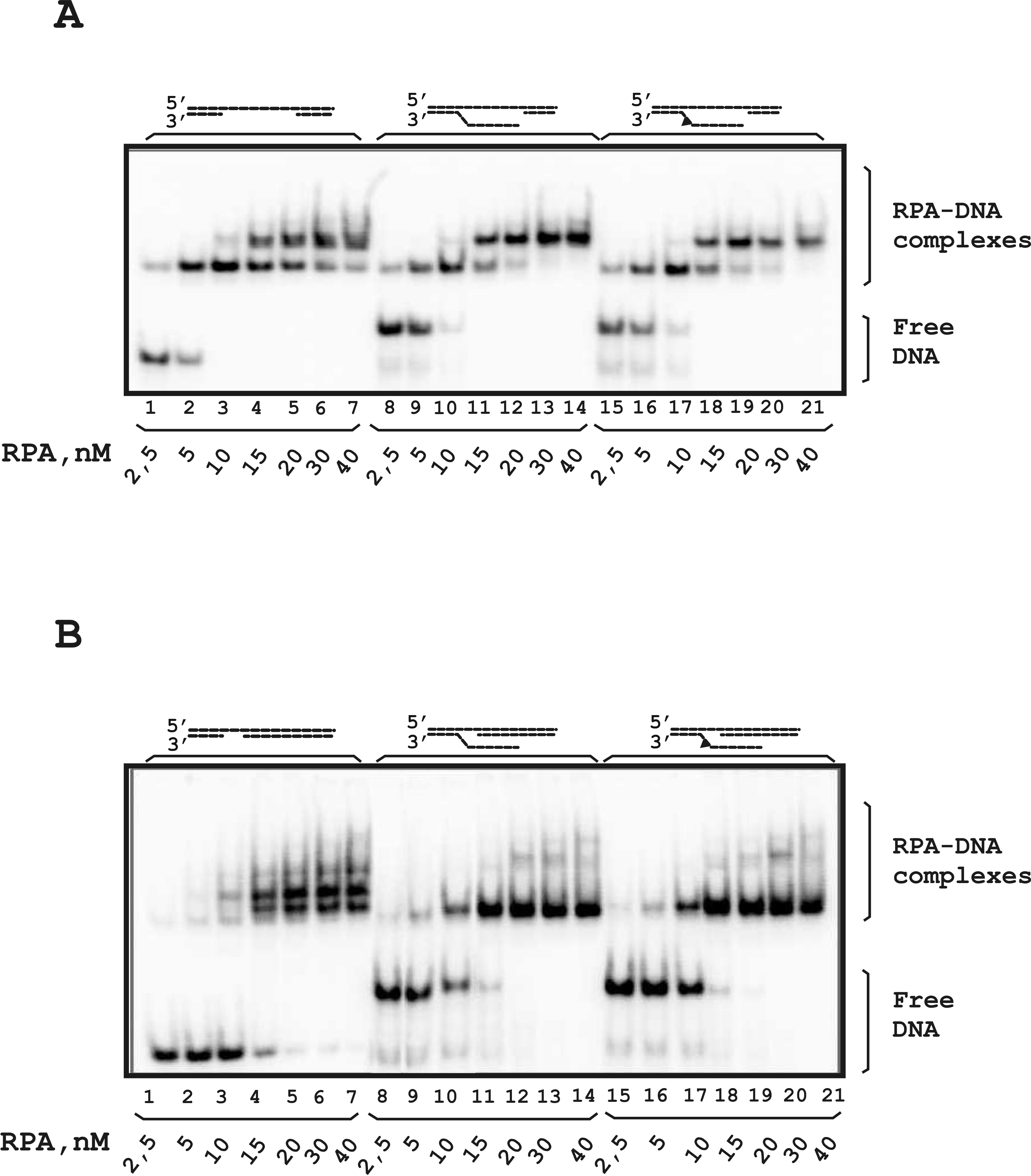

Supplement: S4 Fig — The reaction mixtures (10 μl) contained buffer A, 10 nM 5′-32P-labeled DNA and RPA at the indicated concentrations. A schematic view of the DNA structures is presented at the top: the triangle indicates the position of the bulky lesion. (TIF) [file pone.0190782.s005.TIF]

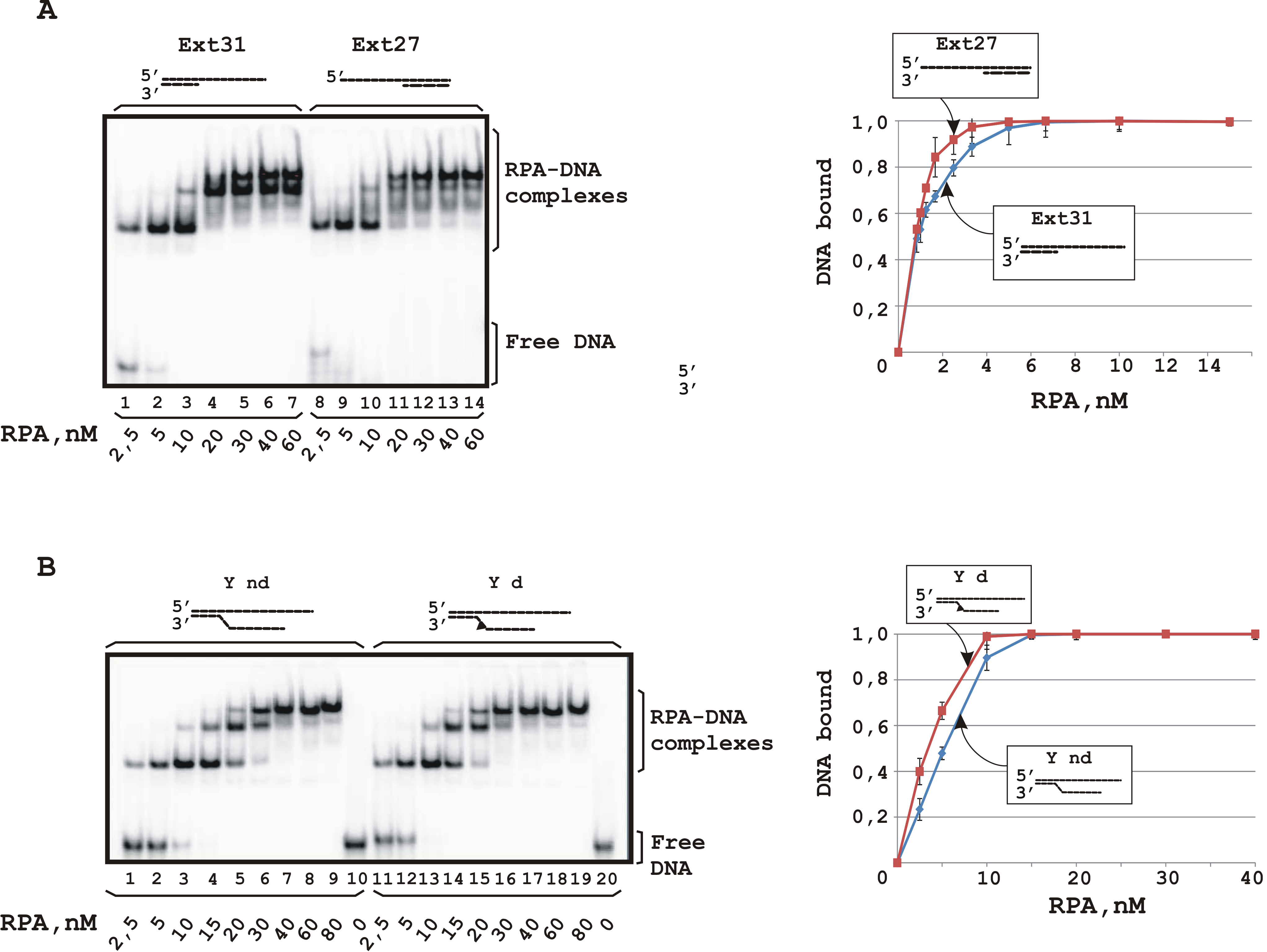

Supplement: S5 Fig — The reaction mixtures (10 μl) contained buffer A, 10 nM 5′-32P-labeled DNA and RPA at the indicated concentrations. A schematic view of the DNA structures is presented at the top: the triangle indicates the position of the bulky lesion. (TIF) [file pone.0190782.s006.TIF]

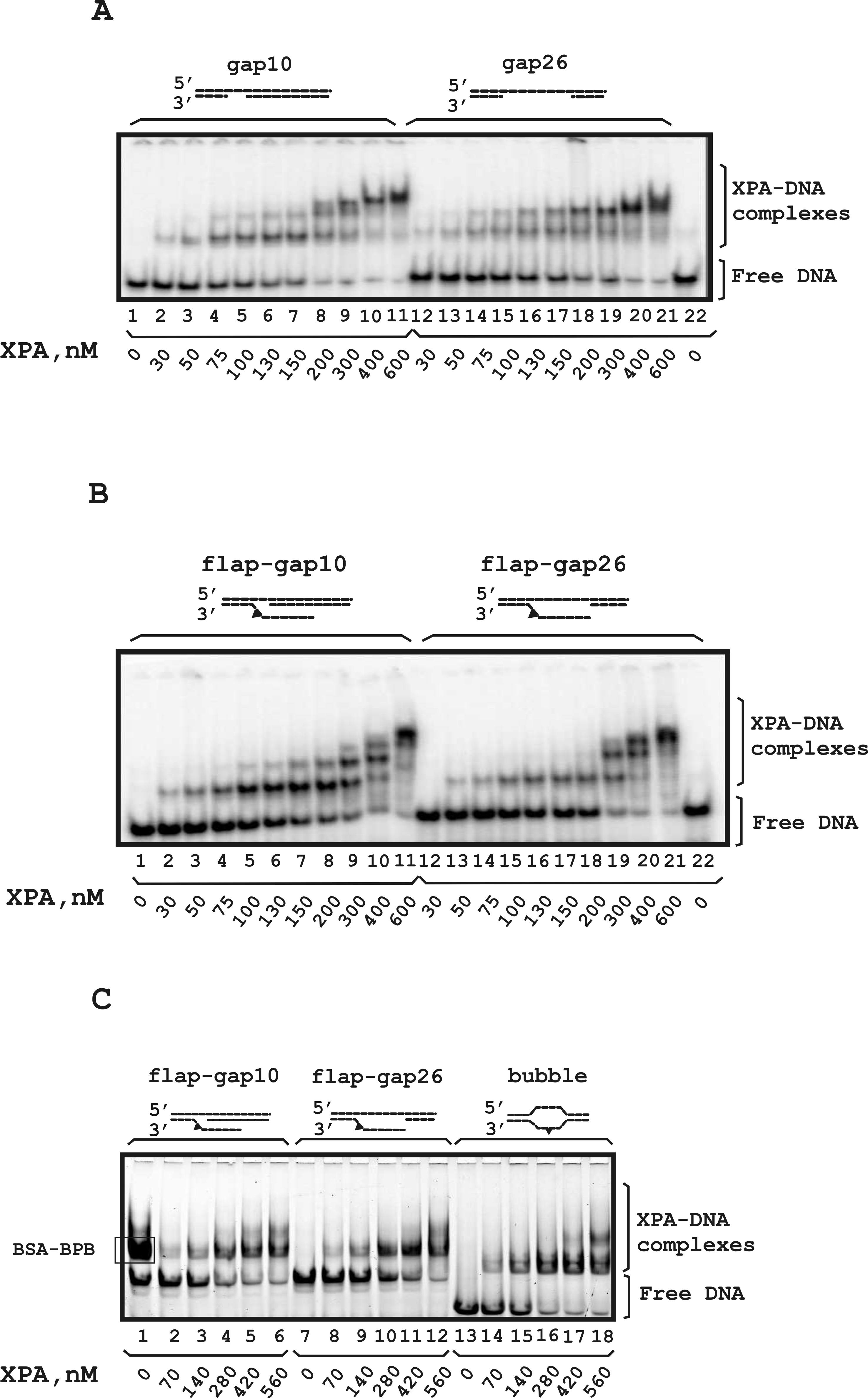

Supplement: S6 Fig — (A) DNA containing a flap and a gap, (B) DNA containing a gap only. The reaction mixtures (10 μl) contained buffer A, 10 nM 5′-32P-labeled DNA and XPA at the indicated concentrations. A schematic view of the DNA structures is presented at the top: the triangle indicates the position of the bulky lesion. (C) XPA binds bubble-DNA more effectively than flap-gap containing DNA. The reaction mixtures (10 μl) contained buffer A, 10 nM Flu-DNA containing flap and 10 nt (lanes 1–6), 26 nt (lanes 7–12) gap or bubble (lanes 13–18) and XPA at the indicated concentrations. In lane 1, the band indicated as BSA-BPB corresponds to a complex of BSA with bromophenol blue that was added to this sample before loading. (TIF) [file pone.0190782.s007.TIF]
